# Case Report: Guillain-Barré syndrome mimicking acute brainstem stroke with severe autonomic dysfunction-complete recovery after early plasma exchange

**DOI:** 10.3389/fphys.2026.1860621

**Published:** 2026-07-16

**Authors:** Jianming Zhu, Li Shu, Yuxuan Peng, Sujun Sun, Chaojun Zhou, Yi Zhong, Ying Li, Xiang Liu, Yanyan Li, Xianglin Liu

**Affiliations:** 1 Department of Neurology, Changde Hospital, Xiangya School of Medicine, Central South University (The First People’s Hospital of Changde City), Changde, China; 2 Department of Nursing, Changde Hospital, Xiangya School of Medicine, Central South University (The First People’s Hospital of Changde City), Changde, China; 3 Department of Radiology, Changde Hospital, Xiangya School of Medicine, Central South University (The First People’s Hospital of Changde City), Changde, China; 4 Department of Electroencephalography and Electromyographym, Changde Hospital, Xiangya School of Medicine, Central South University (The First People’s Hospital of Changde City), Changde, China; 5 Department of Respiratory and Critical Care Medicine, Changde Hospital, Xiangya School of Medicine, Central South University (The First People’s Hospital of Changde City), Changde, China

**Keywords:** autonomic dysfunction, case report, dilated pupils, Guillain-Barré syndrome, plasma exchange, stroke mimic

## Abstract

Acute onset of bilateral ptosis, bulbar palsy, and quadriparesis typically raises suspicion for brainstem infarction. However, when neuroimaging is unrevealing, immune-mediated neuropathies such as Guillain-Barré syndrome (GBS) should be considered. This case highlights the diagnostic challenge and therapeutic response in a seronegative, rapidly progressive GBS variant with severe autonomic involvement. We report a 57-year-old previously healthy man who presented with acute right-sided weakness and dysarthria, progressing over 36 hours to bilateral ptosis, complete ophthalmoplegia, dilated pupils, bulbar palsy, flaccid quadriparesis, urinary retention, and paralytic ileus. Initial brain MRI-DWI was negative. CSF showed no albuminocytologic dissociation. Anti-ganglioside antibodies and neuromuscular junction antibodies were negative. Electromyography revealed motor-predominant polyneuropathy with sympathetic skin response abnormalities. A diagnosis of atypical GBS with severe autonomic involvement was made. Plasma exchange was initiated at 40 hours after onset, followed by a second session on day 5. The patient improved dramatically, walked independently by day 8, was discharged on day 15, and achieved complete recovery at 1 month. This case demonstrates that GBS can present as an acute brainstem stroke mimic with severe ileus and urinary retention, even in the absence of CSF abnormalities or detectable autoantibodies. Early plasma exchange may result in complete neurological recovery. Therefore, clinicians should maintain a high clinical suspicion for immune-mediated neuropathies in patients presenting with rapidly progressive stroke mimics accompanied by autonomic dysfunction.

## Introduction

Guillain-Barré syndrome (GBS) represents a spectrum of acute immune-mediated polyneuropathies, frequently triggered by prior infections and characterized by varying degrees of motor, sensory, and cranial nerve involvement (Shahrizaila et al., 2021). Atypical presentations- particularly those involving prominent cranial nerve deficits or severe autonomic dysfunction-pose diagnostic challenges and may mimic stroke, myasthenia gravis, or botulism (Chen et al., 2021; Sun et al., 2021).

Miller Fisher syndrome (MFS), characterized by ophthalmoplegia, ataxia, and areflexia, is a well-recognized GBS variant (Wakerley et al., 2014; Lee et al., 2024). However, bilateral internal ophthalmoplegia (dilated pupils) and severe autonomic disturbances such as paralytic ileus and urinary retention are exceptionally rare in GBS and are often underrecognized. Furthermore, the absence of cerebrospinal fluid (CSF) albuminocytologic dissociation and negative anti-ganglioside antibody tests early in the disease course do not exclude GBS but may delay treatment (Al-Hakem et al., 2023; Sejvar, 2023).

Herein, we report a patient with an atypical GBS variant that initially mimicked an acute brainstem stroke, presenting with bilateral dilated pupils, complete external ophthalmoplegia, bulbar palsy, quadriparesis, and severe autonomic failure. Despite negative laboratory markers, early plasma exchange resulted in rapid and complete recovery. This case underscores the significance of clinical suspicion and collaborative management in rare GBS phenotypes.

## Case presentation

### Patient information

A 57-year-old man without prior hypertension, diabetes, or family history of neuropathy presented with acute-onset right-sided weakness and dysarthria that began 16 hours earlier while working at home. There was no vaccination history or antecedent infectious disease such as fever or diarrhea. Over the next 10 hours, his left lower limb also became weak, followed by bilateral ptosis and facial droop. A local hospital cranial Computed Tomography (CT) showed bilateral basal ganglia lacunar lesions, but no hemorrhage. He was transferred to our stroke unit.

**Figure 1:**
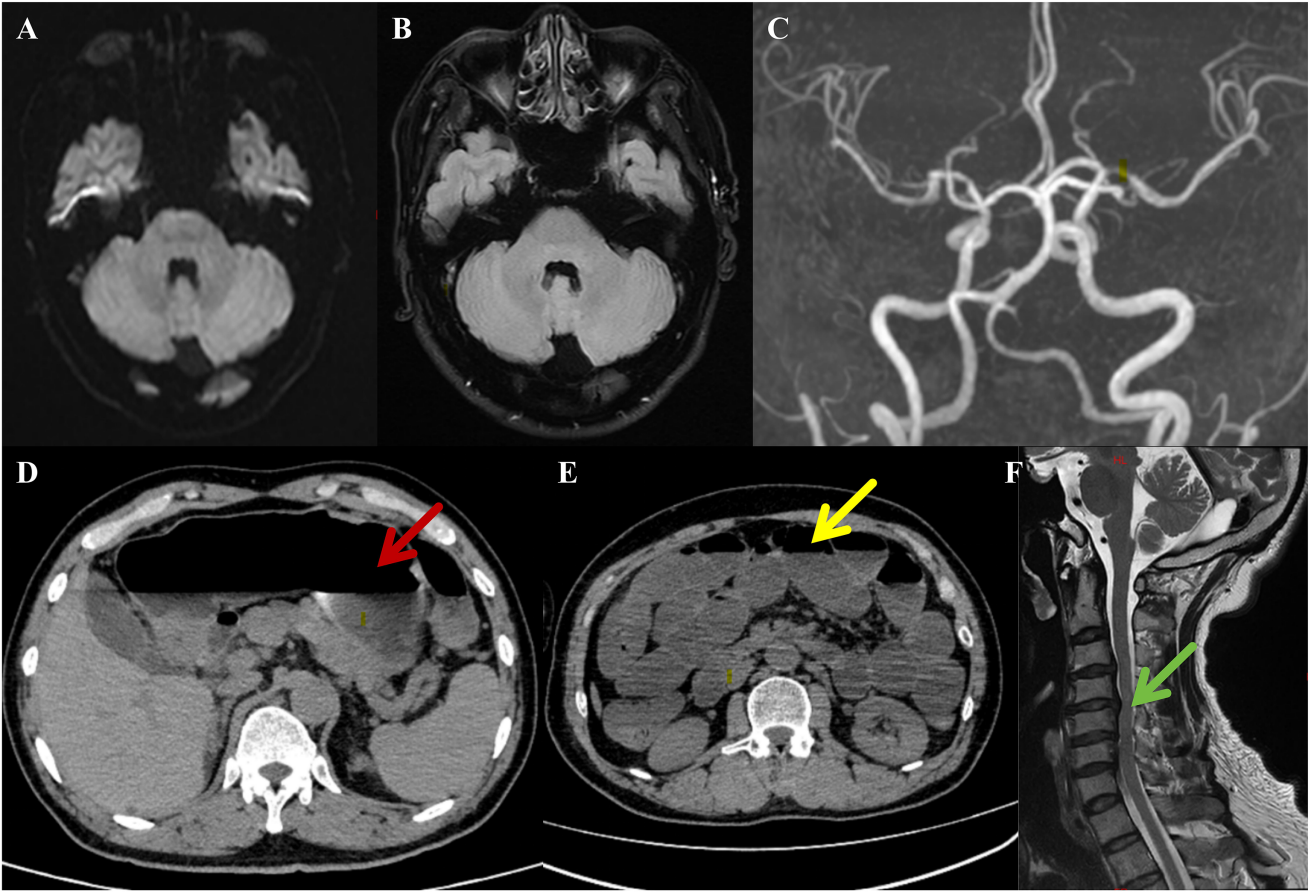
Imaging findings of the patient. Brain magnetic resonance imaging (MRI) showed normalities **(A–C)**. Whole abdominal Computed Tomography **(D, E)**: distension of the gastric lumen (red arrow) and small intestinal loops (yellow arrow), with gas and fluid accumulation, and multiple air-fluid levels observed. Cervical spine T2-weighted MRI **(F)**: C3–7 intervertebral disc bulge and protrusion with C4–6 spinal canal stenosis (green arrow).

On admission (16 hours from onset), he was somnolent with dysarthria. Neurological examination revealed bilateral ptosis, left nasolabial fold flattening, tongue deviation to the left, and diminished gag reflex. Muscle strength was grade 4 in all four limbs with normal tone. Deep tendon reflexes were reduced in the biceps but brisk at the knees, Babinski sign was negative. Cranial CT excluded hemorrhage. Given the time window for endovascular therapy, we recommended CT angiography (CTA)/perfusion (CTP), but the patient declined. He was started on antiplatelet therapy and statin. Brain MRI-DWI (**Figures 1A–C**) performed at 24 hours from onset showed no hyperintensity, effectively excluding brainstem infarction. Cervical spine MRI revealed C3–7 intervertebral disc degeneration and C4–6 spinal stenosis (**Figure 1F**), which may have contributed to the brisk knee reflexes, yet these degenerative spinal changes could not explain the patient’s multiple cranial nerve impairments.

36 hours from onset, his condition deteriorated dramatically. He remained somnolent, developed anarthria, choking on liquids, bilateral dilated pupils (4.5 mm, with sluggish direct and consensual light reflexes), complete external ophthalmoplegia with diplopia (when eyes could be opened), bilateral peripheral facial palsy, and pseudobulbar palsy. Upper limb proximal strength dropped to grade 2 and distally to grade 3; lower limbs remained grade 4. Kubota water swallowing test: grade 5, Hughes Scale grade 4, Medical Research Council (MRC) score 38 points. He also developed urinary retention requiring catheterization, vomiting, abdominal distension, hypoactive bowel sounds, and fecal occult blood positivity. He was placed on nil per os, nasogastric decompression, esomeprazole, somatostatin, and parenteral nutrition. No significant abnormalities were observed in blood pressure, heart rate, sweating, or other autonomic nervous functions. Laboratory and diagnostic findings.

Electrophysiological testing on hospital day 3 (**Supplementary Table 1**) revealed multifocal motor-predominant peripheral neuropathy in all limbs, accompanied by lower limb sensory nerve dysfunction and absent sympathetic skin responses. F waves, needle electromyography and repetitive nerve stimulation (RNS) were unremarkable bilaterally.

Lumbar puncture conducted on hospital day 3 demonstrated normal intracranial pressure; cerebrospinal fluid analysis yielded a protein level of 246 mg/L (reference range, 80–430 mg/L) and a cell count of 1 × 10^6^/L (reference range, 0–10 × 10^6^/L), with no evidence of albuminocytologic dissociation.

Serum anti-ganglioside IgG and IgM antibodies (Sulfatide, GM1, GM2, GM3, GM4, GD1a, GD1b, GD2, GD3, GT1a,GT1b, GQ1b) were negative. A panel of neuromuscular junction antibodies (acetylcholine receptor, muscle-specific kinase, low-density lipoprotein receptor-related protein 4, titin, and ryanodine receptor) was negative.

Additional findings during hospitalization included: A non-contrast abdominal CT (**Figures 1D, E**) showed distended stomach and small intestine with air and fluid, and multiple air-fluid levels, suggestive of paralytic ileus. Among the electrolytes, serum potassium was normal, ruling out hypokalemia as the etiology. elevated inflammatory markers (IL-6 11.22 pg/ml, IL-8 75.03 pg/ml) with low-grade fever; right upper extremity and bilateral calf vein thromboses on vascular ultrasound, serial D-dimer levels initially increased (7.14 mg/L) then decreased (3.42 mg/L), and persistently low white blood cell (3.21 × 10^9^/L) and lymphocyte counts (0.59 × 10^9^/L) that normalized after discharge.

### Clinical course

Based on the clinical course, a diagnosis of atypical GBS with severe autonomic and cranial nerve involvement was made (Fokke et al., 2014). After 40 hours from onset, the patient received plasma exchange (PE) (1600 mL).

On day 3, bilateral ptosis improved, and upper limb strength increased to 3/5 proximally and 4/5 distally. A second PE (1600 mL) was performed on day 5. On day 6, he was fully conscious, dysarthria had improved, ocular movements had normalized, though bilateral facial palsy persisted. Upper limb strength was grade 4 proximally and grade 5 distally; lower limbs were grade 5. On day 8, he could walk several hundred meters independently. Pupils remained equal at 4 mm with sluggish reflexes. Bowel and bladder functions normalized, and he resumed oral feeding. He was discharged on day 15, Hughes Scale grade 2, MRC score 58 points. At one-month follow-up, he had completely recovered, with normal blood counts and biochemistry.

**Figure 2:**
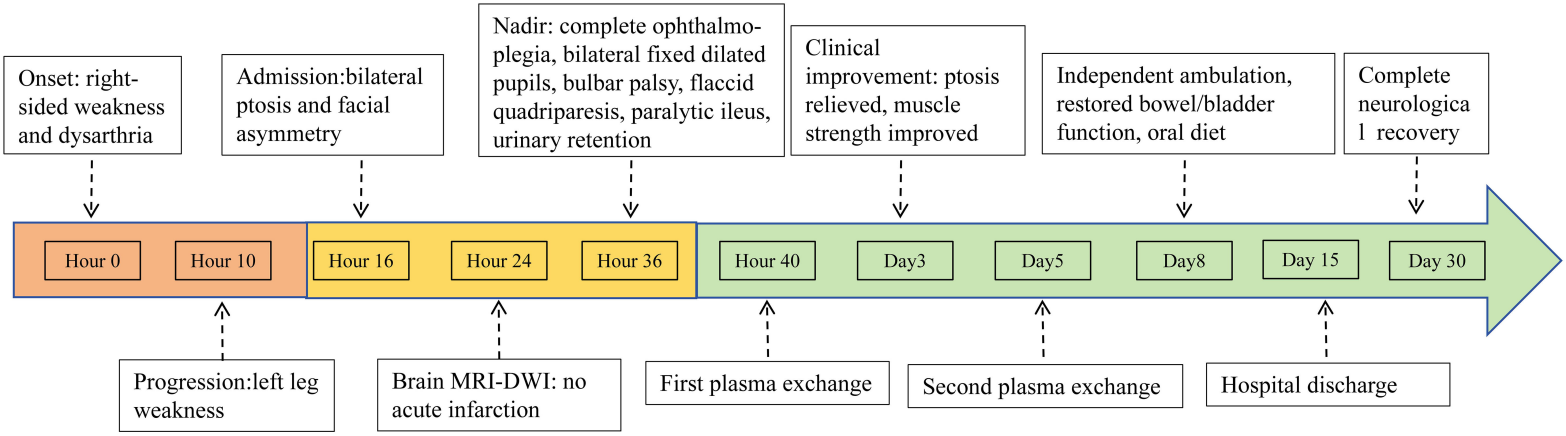
Clinical timeline from symptom onset to complete recovery. MRI-DWI, magnetic resonance imaging-diffusion weighted imaging.

A schematic timeline summarizing the clinical course, diagnostic workup, treatment, and outcome of the patient is presented in **Figure 2**.

### Patient perspective

During the acute phase, the patient experienced extreme anxiety and fear due to complete bilateral ptosis with visual obstruction, combined with an inability to move or speak. The nursing and medical team provided continuous bedside reassurance and played audio recordings from his family, which greatly alleviated his distress and improved cooperation with treatment. He expressed profound gratitude for the multidisciplinary care and hopes that sharing his story will help others with similar rare presentations.

## Discussion

We describe a patient with an atypical GBS variant characterized by: (i) acute bilateral internal and external ophthalmoplegia (dilated pupils), (ii) severe autonomic dysfunction including paralytic ileus and urinary retention, (iii) rapid progression to nadir within 36 hours, (iv) absence of CSF albuminocytologic dissociation and negative anti-ganglioside antibodies, and (v) dramatic response to PE.

Dilated pupils are an exceptional manifestation of GBS. They imply involvement of the ciliary ganglion or postganglionic parasympathetic fibers, which are unmyelinated and can be affected in severe immune-mediated autonomic neuropathies (Zaeem et al., 2019). To our knowledge, only a few GBS cases with bilateral internal ophthalmoplegia have been reported, often in the setting of anti-GQ1b antibody-positive MFS (Ye et al., 2026). Notably, our patient was seronegative for anti-GQ1b and common ganglioside antibodies. This may result from early testing at 36 hours with low titers or unidentified ganglioside antibodies.

Severe autonomic dysfunction, including paralytic ileus and urinary retention, is underrecognized in GBS but can be life-threatening (Zaeem et al., 2019). Our patient’s abnormal sympathetic skin response confirmed autonomic nerve fiber involvement. The rapid recovery of bowel and bladder function after PE supports an immune-mediated pathogenesis.

The absence of albuminocytologic dissociation early in the course (day 3) does not exclude GBS, as up to 44% of patients have normal CSF protein on day 3 (Jacobs et al., 2017; Sejvar, 2023). Similarly, negative anti-ganglioside antibodies are not uncommon, especially in pure motor or pharyngeal-cervical-brachial variants (Hughes, 2024). The diagnosis in our case relied on clinical progression, electrodiagnostic evidence of acute motor-predominant polyneuropathy, and exclusion of mimics (Fokke et al., 2014).

Our patient’s initial presentation with asymmetric weakness and dysarthria mimicked a stroke, as previously highlighted by Sun et al (Sun et al., 2021). However, the rapid development of bilateral ptosis, ophthalmoplegia, and pupils, along with a negative MRI, steered the diagnosis away from vascular etiology. This patient had no relevant epidemiological history, and no co-diners developed symptoms, ruling out botulism (Silva Campos et al., 2023). This patient had no diurnal variation, normal RNS findings, and negative neuromuscular junction antibodies, excluding myasthenia gravis (Chen et al., 2021). Multiple sclerosis (MS) was also considered but deemed unlikely: brain and cervical spinal MRI revealed no demyelinating lesions, the patient had no prior history of demyelinating episodes, and his clinical manifestations failed to satisfy the criteria for dissemination in time and space required for MS diagnosis (Marcus, 2022). Although cerebrospinal fluid oligoclonal band testing was not performed in this case, the combination of negative MRI findings and the absence of clinical dissemination effectively excluded MS as a diagnostic consideration.

According to the 2023 European Association of Neurology and the Peripheral Nerve Society (EAN/PNS) guideline on GBS, only PE and intravenous immunoglobulins (IVIG) have proven efficacy as immune treatments in the acute stage (van Doorn et al., 2023; Wiegers and Jacobs, 2025). Several factors favored PE over IVIG in this patient. First, the patient was at high risk for IVIG-related thromboembolic events given markedly elevated D-dimer levels and documented venous thromboses, whereas PE does not confer this risk (Wang et al., 2024). Second, a recent meta-analysis demonstrated that PE has lower overall healthcare costs compared with IVIG in autoimmune neurological disorders (Kimber et al., 2026). Third, PE was readily available at our institution as a regional referral center for neuroimmunological disorders, and the treatment plan was established through multidisciplinary discussion and shared decision-making, incorporating the patient’s informed preference and written consent. The excellent response to PE aligns with the current recommendation that early immunotherapy improves outcomes in severe GBS (Chevret et al., 2017). Case reports have shown that for patients who respond poorly to PE/IVIG therapy, Add-On treatment with efgartigimod can be an option and may also lead to a favorable prognosis (Ripellino et al., 2025).

This case also underscores the value of multidisciplinary collaboration. The involvement of neurology (stroke and neuroimmunology teams), critical care, general surgery (for ileus), hematology (for leukopenia), Vascular Surgery(for thrombosis), nutrition, rehabilitation, and transfusion medicine Department was essential for managing complications and achieving a favorable outcome.

Limitations: Due to the patient’s limited financial resources, we were unable to perform serial CSF examinations or nerve conduction studies after recovery, and anti-nodal/paranodal antibodies were not tested. However, the clinical presentation and treatment response are consistent with an autoimmune neuropathy.

## Conclusion

We report a rare case of atypical GBS presenting with bilateral dilated pupils, complete external ophthalmoplegia, severe autonomic failure (paralytic ileus, urinary retention), and rapid progression mimicking a brainstem stroke. Despite negative CSF and serological markers, early PE was associated with rapid and complete recovery. This case expands the phenotypic spectrum of GBS and reminds clinicians to consider this diagnosis in acute flaccid paralysis with prominent cranial and autonomic involvement, even when typical laboratory features are absent.

## Data Availability

The original contributions presented in the study are included in the article/**Supplementary Material**. Further inquiries can be directed to the corresponding author.
