# Supplementary material for: Case Report: Guillain-Barré syndrome mimicking acute brainstem stroke with severe autonomic dysfunction-complete recovery after early plasma exchange
Source: Front Physiol. 2026 Jul 16;17:1860621. doi: 10.3389/fphys.2026.1860621 (PMC13421435; doi:10.3389/fphys.2026.1860621)
Supplement: Supplementary file 1 [file Table1.docx]

**Table1** Motor nerve conduction study results ( electromyography)

| Tested nerve | Latency | Amplitude | Conduction velocity |
| --- | --- | --- | --- |
|  | (ms) | (μV) | (m/s) |
| Left ulnar nerve |  |  |  |
| Wrist | 1.83（≤3.1） | 5.1（≥7.0）↓ |  |
| Elbow | 6.5 | 4.4（≥7.0）↓ | 55.7（≥51.0） |
| Right ulnar nerve |  |  |  |
| Wrist | 2.25（≤3.1） | 5.3（≥7.0）↓ |  |
| Elbow | 6.77 | 4.8（≥7.0）↓ | 56.4（≥51.0） |
| Left median nerve |  |  |  |
| Wrist | 2.68（≤4.2） | 2.4（≥7.0）↓ |  |
| Elbow | 6.9 | 2.3（≥7.0）↓ | 61.6（≥50.0） |
| Right median nerve |  |  |  |
| Wrist | 2.86（≤4.2） | 3.3（≥7.0）↓ |  |
| Elbow | 6.92 | 3.3（≥7.0）↓ | 55.4（≥50.0） |
| Left tibial nerve |  |  |  |
| Ankle | 3.9（≤5.8） | 3.0（≥5.0）↓ |  |
| Knee | 11.3 | 2.4（≥5.0）↓ | 51.4（≥39.4） |
| Right tibial nerve |  |  |  |
| Ankle | 3.85（≤5.8） | 2.3（≥5.0）↓ |  |
| Knee | 10.9 | 1.39（≥5.0）↓ | 53.9（≥39.4） |
| Left common peroneal nerve |  |  |  |
| Ankle | / | No potential | / |
| Fibular head | / | No potential | / |
| Right common peroneal nerve |  |  |  |
| Ankle | 4.36（≤4.6） | 0.59（≥3.0）↓ |  |
| Fibular head | 11.1 | 0.59（≥3.0）↓ | 44.5（≥39.8） |

Motor nerve conduction studies of the upper and lower limbs showed decreased amplitudes in the bilateral ulnar, bilateral median, bilateral tibial, and right common peroneal nerves, while no motor response was elicited in the left common peroneal nerve. Electrophysiological findings consistent with multifocal peripheral neuropathy, predominantly involving the motor nerves of all four limbs.
